# Comparison of the inactivation of seven foodborne pathogens and spoilage bacteria under 405 nm blue light treatment in liquid media and on solid surfaces

**DOI:** 10.1128/spectrum.00093-25

**Published:** 2025-05-23

**Authors:** Minji Hur, Francisco Diez-Gonzalez

**Affiliations:** 1 Center for Food Safety and Department of Food Science and Technology, University of Georgia 308511, Griffin, Georgia, USA; Agriculture and Agri-Food Canada, Lacombe, Canada

**Keywords:** blue light, foodborne pathogens, food safety, *Lactobacillus*, *Pseudomonas*

## Abstract

Antimicrobial blue light (aBL; 405 nm) can reduce the viability of multiple bacteria, but there are few reports comparing the susceptibility of foodborne bacteria when exposed to similar conditions. This study was undertaken to evaluate the efficacy of aBL against foodborne pathogens and spoilage bacteria when treated in liquid media and on different surfaces. Cocktails of *Listeria*, *Salmonella*, *Escherichia*, *Pseudomonas*, *Cronobacter*, *Lactobacillus*, and *Staphylococcus* were incubated in tryptic soy broth (TSB) and phosphate-buffered saline (PBS), as well as on stainless steel (SS) coupons, avocados, and cherry tomatoes as dried cells. Reductions in cell viability were determined after exposure to 405 nm aBL at two temperatures. aBL doses for liquid, SS coupons, and fruits were 1,512, 1,739, and 1,944 J/cm^2^, respectively. Microbiological counts were determined using standard complex media, and Analysis of variance (ANOVA) was used to determine significant differences (*P* < 0.05). The viability of *Pseudomonas* in PBS was reduced by more than 8 Log CFU/mL of aBL exposure, but the reduction of other bacteria was less than 4 Log CFU/mL. In TSB, more than 5 Log CFU/mL were inactivated in all genera. Reductions in viable *Cronobacter* and *Salmonella* dried cells on SS coupons were less than 2 Log CFU/coupon. Treatment of cells on avocados resulted in small reductions of *Cronobacter* and *Salmonella* (<1.5 Log CFU/skin spot) at 20°C compared to other species. These findings suggest that the susceptibility of different bacterial genera may be quite variable in response to aBL treatment, and these differences should be considered for potential applications.

**IMPORTANCE:**

Ready-to-eat (RTE) foods have been associated with multiple outbreaks caused by foodborne pathogens. While the safety of many RTE foods such as fresh produce relies on traditional cleaning and sanitation systems, they are often insufficient to keep bacteria off food-contact surfaces within the processing and packing environment. Antimicrobial blue light (aBL) in the range of 400–470 nm of wavelength may be a promising disinfectant alternative. However, more comprehensive studies are needed to better assess the effectiveness of aBL against a wide range of foodborne pathogens and spoilage. Such research could provide valuable insights into aBL’s potential as a crucial tool for ensuring food safety.

## INTRODUCTION

Ready-to-eat (RTE) foods such as fresh produce, fruits, and deli meats pose a significant food safety challenge due to minimal processing and their susceptibility to being contaminated with foodborne pathogens (1, 2). The safety of RTE foods is dependent on the use of effective cleaning and sanitation (3). Unfortunately, existing systems such as chemical sanitizers are often insufficient to adequately control foodborne pathogens, particularly on hard-to-reach surfaces that could harbor these microbes and facilitate their transfer to RTE food during processing and packaging (4). This shortfall stresses the importance of developing novel supplemental intervention strategies for food processing and packaging environments.

Blue light produced by light-emitting diodes (LEDs) in the range of 400–470 nm wavelength is a type of electromagnetic radiation that has emerged as a promising technology with multiple applications (5). Blue light has been commercially used in 3D printing for curing and as a treatment for inflammatory acne (6, 7). Blue light has also been investigated to evaluate its efficacy to contribute to the cultivation and quality of lettuce leaves and strawberries (8–10). In addition to these applications, extensive evidence shows that exposure to blue light can inactivate various species of bacterial cells. Due to its antimicrobial effect, numerous reports have documented the killing of diverse bacterial genera.

Antimicrobial blue light (aBL) offers several advantages over traditional methods, including affordability and less risk to operators compared to conventional UV light (11). UV light has traditionally served as a surface disinfectant for multiple applications, such as hospital settings (12). UV light (wavelength 240–400 nm) is limited by its low penetration and potential to harm human skin and eyes. The implementation of aBL in food facilities may offer an alternative for controlling foodborne pathogens and spoilage bacteria. Guffey and collaborators successfully inactivated *Escherichia coli* and *Salmonella* on processed meat and cucumbers, respectively (13). aBL was also reported to reduce *Salmonella* viability in orange juice (14).

While the exact mode of action of aBL remains uncertain, it is believed to be mediated by the general bacterial response to visible light (15). One proposed mechanism involves endogenous porphyrin compounds within the bacterial cell (16). Upon exposure to aBL in the presence of oxygen, these porphyrins absorb light with the subsequent production of reactive oxygen species (ROS). These ROS, including superoxide ions, can react with essential cellular components such as nucleic acids, lipids, and proteins, ultimately causing bacterial cell death. Recent research suggested that it is unlikely that gram-negative bacteria develop resistance to aBL (17).

Previous research has explored the efficacy of aBL against hospital-acquired pathogens. For instance, Halstead’s group evaluated 405 nm blue light treatments against nine nosocomial wound pathogens in suspensions and biofilms (18). Some studies demonstrated the effectiveness of 405 nm aBL on specific foodborne pathogens on surfaces and media relevant to food processing facilities (19). Ghate and other researchers compared different foodborne pathogens under 465, 521, and 642 nm of visible light with a maximum dosage of 688 J/cm^2^ (20). Furthermore, our research group recently observed significant inactivation of *Listeria monocytogenes* on stainless steel (SS) coupons under aBL treatments (21).

aBL at 405 nm has been proposed against food-related bacteria as an alternative intervention technology. For instance, the antimicrobial effect of 405 nm blue light was investigated on major gram-positive foodborne pathogens at refrigeration temperature (22). A more comprehensive comparison study was conducted by Murdoch’s group, in which single strains of *Salmonella*, *Shigella*, *Escherichia*, *Listeria*, and *Mycobacterium* in phosphate-buffered saline (PBS) were exposed to 405 nm aBL (23). Cocktails of *L. monocytogenes* on enoki mushrooms were inactivated when treated with a combination of 405 nm aBL and lactic acid (11). More recently, *E. coli* O157:H7 and *L. monocytogenes* on several food-contact surfaces used in food processing plants were effectively reduced by 405 nm aBL (24).

A systematic comparison of the susceptibility of multiple foodborne pathogens and spoilage bacteria to aBL under different conditions is largely missing in the literature, and this study was undertaken to identify differences among bacterial genera. The goal of the study was to compare the effectiveness of 405 nm aBL treatments against *L. monocytogenes*, *Salmonella* Typhimurium, *Pseudomonas*, Shiga toxin-producing *E. coli*, *Cronobacter sakazakii*, *Staphylococcus aureus*, and *Lactobacillus*, as suspended cells in standard buffer and complex media, and as dried cells on stainless steel and two representative food surfaces: avocados and cherry tomatoes.

## RESULTS

### aBL treatment in liquid media

**Fig 1:**
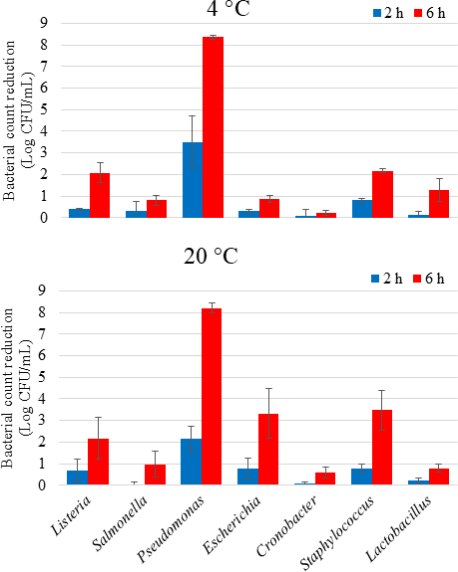
The effect of blue light exposure (405 nm) on the viability of cell suspension of different bacterial genera in PBS. Cocktails of 4–5 strains per genera were treated with total doses of 504 and 1,512 J/cm^2^ after 2 and 6 h, respectively.

In PBS, the initial counts (7.4–8.6 Log CFU/mL) of all cocktails of seven foodborne and spoilage bacterial species were gradually reduced during 6 h at 4 and 20°C by exposure to aBL (Fig. 1). After 2 h, the reduction of all bacteria except for *Pseudomonas* was less than 1 Log CFU/mL at both temperatures compared to the pre-exposure initial counts. After 6 h, the *Pseudomonas* viable count was diminished by more than 8 Log CFU/mL, which was more than double the reduction observed in *Escherichia* and *Staphylococcus* cell suspensions (*P* < 0.05). The viability of *Salmonella* and *Cronobacter* suspensions declined very little (less than 1 Log CFU/mL) at both temperatures. The losses of *Listeria* viability were very similar at both temperatures (*P* > 0.05).

**Fig 2:**
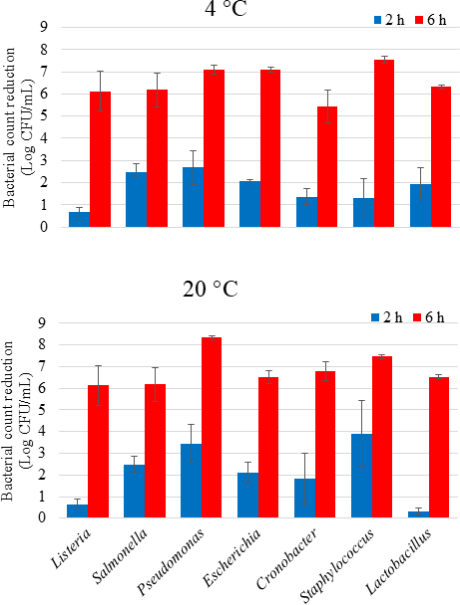
The effect of blue light exposure (405 nm) on the viability of TSB cultures of different bacterial genera. Cocktails of 4–5 strains per genera were treated with total doses of 504 and 1,512 J/cm^2^ after 2 and 6 h, respectively.

The initial bacterial counts in tryptic soy broth (TSB) were between 7.16 and 8.76 Log CFU/mL for all seven species. After treatment with 504 J/cm^2^, the viability reduction ranged from 0.5 Log CFU/mL for *Listeria* at 4°C to 3.9 Log CFU/mL for *Staphylococcus* at 20°C (Fig. 2). Six hours of aBL exposure (1,512 J/cm^2^) resulted in viability reductions greater than 5 and 6 Log CFU/mL at 4°C and 20°C, respectively. In TSB, the *Staphylococcus* cultures had the largest inactivation of 7.56 Log CFU/mL compared to all other bacteria with the exception of *Pseudomonas* (*P* < 0.05).

### aBL treatment on stainless steel as dried cells

**Fig 3:**
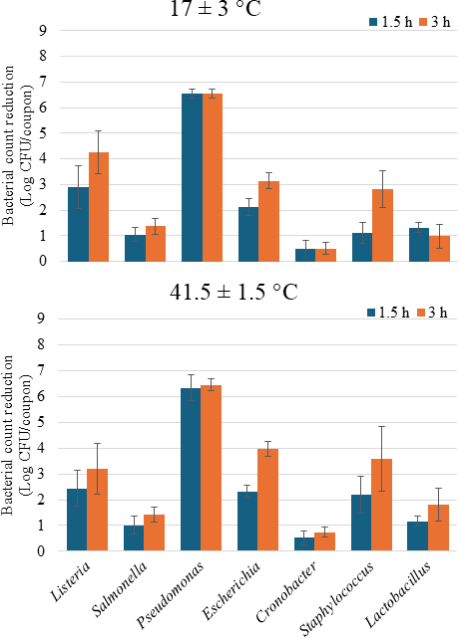
The effect of blue light exposure (405 nm) on the viability of cells dried on SS coupons of different bacteria genera. Cocktails of 4–5 strains per genera were treated with total doses of 869.4 and 1,738.8 J/cm^2^ after 1.5 and 3 h, respectively.

Cells (7.6 Log CFU/coupon) inoculated and dried on SS coupons were treated with aBL for 3 h at 17°C and 41.5°C (Fig. 3). After 1.5 h of exposure (869.4 J/cm^2^), the viable counts of *Pseudomonas* declined more than 6 Log CFU/coupon—more than twice the reduction observed for the rest of the bacteria (*P* < 0.05) at both temperatures—compared to the initial counts. Reductions of less than 1 Log CFU/coupon were observed when *Cronobacter* cells were treated at both doses and temperatures, and this result was different from those of other bacteria (*P* < 0.05). The reductions of *Salmonella* and *Lactobacillus* were less than 2 Log CFU/coupon after 1,738.8 J/cm^2^ aBL exposure.

### aBL treatment on fruit surfaces

**Fig 4:**
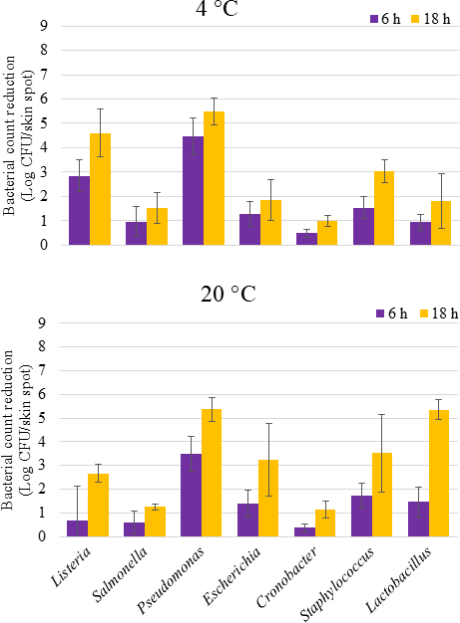
The effect of blue light exposure (405 nm) on the viability of cells of different bacteria genera dried on avocado surfaces. Cocktails of 4–5 strains per genera were dried overnight on avocado skins treated with total doses of 648 and 1,944 J/cm^2^ after 6 and 18 h, respectively.

Exposure to aBL at both temperatures reduced the viability of bacterial species on avocado surfaces variably (Fig. 4). Compared to the initial counts, aBL treatments (648 J/cm^2^) resulted in less than 2 Log CFU/skin spot of viable reductions of all bacteria except for *Pseudomonas* at 20°C and *Pseudomonas* and *Listeria* at 4°C. After exposure to 1,944 J/cm^2^, the viability of *Pseudomonas* decreased by more than 5 Log CFU/skin spot at both temperatures. The viability of *Lactobacillu*s and *Listeria* cells was also markedly reduced at this level, surpassing 5 and 4.5 Log CFU/skin spot at 20°C and 4°C, respectively. In contrast, *Salmonella* and *Cronobacter* had a less pronounced response to aBL treatment, as none of their inactivation results were larger than 1.5 Log CFU/skin spot after 18 h aBL treatment.

**Fig 5:**
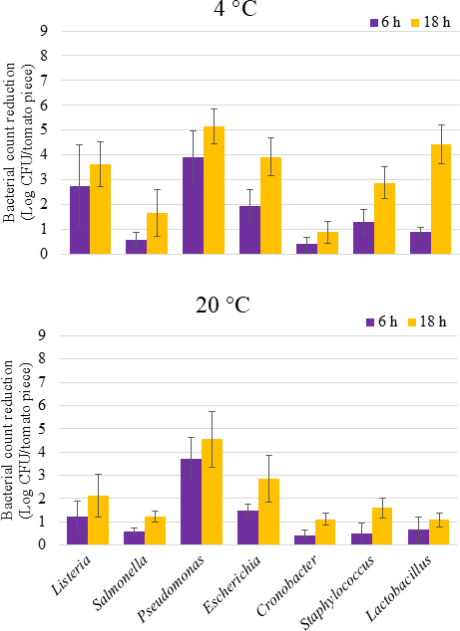
The effect of blue light exposure (405 nm) on the viability of cells of different bacteria genera dried on surfaces of cherry tomatoes. Cocktails of 4–5 strains per genera were dried overnight and treated with total doses of 648 and 1,944 J/cm^2^ after 6 and 18 h, respectively.

The antibacterial efficacy of aBL at 405 nm against dried bacterial cells on cherry tomatoes was evaluated at both temperatures for up to 1,944 J/cm^2^ (Fig. 5). The inactivating effect of aBL was enhanced by incubating at 4°C for all bacteria, but the difference with 20°C was only significant for *Staphylococcus* and *Lactobacillus* (*P* < 0.05). At 20°C, the reduction was less than 1.6 Log CFU/tomato piece after at 1,944 J/cm^2^ for *Salmonella*, *Cronobacter*, *Staphylococcus*, and *Lactobacillus*. At 4°C, the viabilities of *Salmonella* and *Cronobacter* were impacted by less than 2 Log CFU/tomato piece at the same doses.

## DISCUSSION

aBL has emerged as a promising microbial control intervention strategy for potential use in the food industry. It is uncertain whether most bacteria are equally susceptible to aBL because of the limited number of studies of side-by-side comparisons of the effectiveness of aBL against diverse food-related bacteria. To address this research gap, we investigated the efficacy of 405 nm aBL treatment against seven food-related bacterial genera. This study involved different test matrices, including liquid media, stainless steel surfaces, and the surfaces of avocados and cherry tomatoes. The observed differences in susceptibility among bacterial species could be attributed to several factors, including temperature, growth environment, and physiological state of bacteria. These differences illustrated the complexity of the aBL’s antimicrobial mechanism.

The presence of nutrients is crucial for bacterial growth and survival (25), and a rich medium like TSB provides an excess of nutritional compounds. An interesting observation in this study was a large reduction when bacteria were incubated in TSB and treated with aBL at two temperatures. This result was markedly different from that obtained with PBS as the liquid media. In this latter case, only the *Pseudomonas* counts were reduced by more than 7 Log CFU/mL, while other bacteria had no more than 2 Log CFU/mL viability losses. This unique finding led us to hypothesize that bacteria initiated growth in rich media as a response to nutrient availability, and this growth activation increased their susceptibility to aBL stress. An RNA-seq-based study confirmed that the exponential phase cells of *L. monocytogenes* were more vulnerable to aBL (470 nm) than stationary cells (26). Furthermore, Abana et al. (27) investigated the efficacy of aBL throughout different bacterial growth phases, concluding that cells in the exponential phase of *E. coli* strain MG1655 and enterotoxigenic strain E9034A were more susceptible to 455 nm of aBL than the stationary phase cells. These reports supported our hypothesis that the increased susceptibility of TSB-suspended cells was because they were in an exponential phase.

The irradiance doses (J/cm^2^) used in this study were determined by multiplying the average light intensity (W/cm^2^) by the treatment time (s) (28). Previous research has indicated that total irradiation dose determines the effectiveness of aBL, but it is critical that studies report the actual exposure dose in Joules per square centimeter. Some studies only disclose the lamp’s nominal intensity or the distance, which is not sufficient to calculate the exposure dose. The omission of that information limits the reproducibility and comparison of findings (11, 29). This knowledge can contribute to optimizing aBL applications in various food processing and fresh produce packing plants.

The maximum total dosages used in this study in liquid, SS coupons, and fruit surfaces were 1,512, 1,739, and 1,944 J/cm^2^, respectively. Most studies have used a relatively low dosage of less than 1 kJ/cm^2^ of blue light treatment against food-related bacteria. The highest dosage with 405 nm aBL in other studies was in the range of 288 J/cm^2^ (23) for liquid and 576 J/cm^2^ (30) for agar surfaces. A dose of 1.7 kJ/cm^2^ (405 ± 5 nm) blue light resulted in reductions of approximately 1 Log CFU/cm^2^ of *Salmonella* inoculated on fresh-cut papaya at refrigerated temperatures (30). This finding is similar to our results from experiments of *Salmonella* inoculated on avocados and cherry tomatoes that were reduced between 1 and 2 Log CFU/spot, after exposure to 1,944 J/cm^2^. In contrast, more than 6 Log CFU of *Salmonella* viable counts were reduced after 405 nm aBL treatment with 665 J/cm^2^ on stainless steel as dry cells (31), but in our experiments, we determined only 1 Log CFU reduction (869.4 J/cm^2^) of *Salmonella* dried cells with a light device of similar intensity. This difference in killing can be simply explained by the exposure temperature of 94°C or higher reported in that study, compared to our experimental temperature that was never greater than 43°C. The experimental design used by Minor and Sabillón (31) used a high-intensity lamp that generated heat and a treatment chamber that must have prevented any cooling of the exposed coupon. The same authors reported that the type of heat produced by the high-intensity lamp could have played a role in killing bacterial cells in addition to the aBL inactivation.

This study evaluated cocktails of two gram-positive and five gram-negative bacteria, including foodborne pathogens and spoilage bacteria (Table 1). Our results suggested that the sensitivity to aBL of gram-positive and gram-negative bacteria was variable. Among gram-negative bacteria, *Salmonella* and *Cronobacter* were the most tolerant to aBL under most experimental conditions, and *Pseudomonas* was the most sensitive organism. The two gram-positive bacteria tested, *Listeria* and *Staphylococcus*, had less variation in their response to aBL. Other researchers have reported that there could be a difference between gram-positive and gram-negative bacteria susceptibility and pointed out the need for examination of multiple gram-positive and gram-negative bacteria (20, 24). Thus, the susceptibility of bacteria to aBL appears to be linked to intrinsic bacterial characteristics rather than to the presence of the outer membrane.

**TABLE 1:** List of strains that were used in this study by species, serovar, strains, and source^*a*^

Genus	Species	Serovar/serotype	Strain	Source^*b*^
*Listeria*	*monocytogenes*	4b	ATCC 19115	Human
		4b	19117	Sheep
		4b	Coleslaw	Coleslaw
		4b	G1091	Coleslaw outbreak
		1/2a	2011L-2626	Cantaloupe outbreak
*Salmonella*	*enterica*	Typhimurium	ATCC 14028	
		Enteritidis	PT30	Almond outbreak
		Heidelberg	SD67	Turkey
		Newport	115910-K	Beef
		St. Paul	E2008001236	2008 jalapeno pepper outbreak
*Escherichia*	*coli*	O157:H7	ATCC 43895	Raw hamburger meat
		O157:H7	K3995	2006 spinach outbreak
		O157:H7	K4492	2006 Taco Bell lettuce outbreak
		O157:H7	SEA13B88	Unpasteurized apple cider
*Pseudomonas*	*fluorescens*	NA	NCTC 10038	
	*aeruginosa*	NA	NCTC 10332	
		NA	ATCC 27853	
		NA	ATCC 15442	
*Cronobacter*	*sakazakii*	NA	TN1988	Baby formula
		NA	3439	Food isolate
		NA	2855	Clinical isolate
		NA	CDC 1123-79	Blood of newborn
		NA	3437	Food isolate
*Lactobacillus*	*fermentum*	NA	ATCC 14931	
		NA	36	NA
	*plantarum*	NA	CaTC2	Animal-derived foodstuff
		NA	2234	NA
	*bavaricus*	NA	LB5	NA
*Staphylococcus*	*aureus*	NA	ATCC 6538	Human lesion
		NA	ATCC 27664	Chicken tetrazzini
		NA	ATCC 13565	Ham involved in food poisoning
		NA	ATCC 25923	Clinical isolate
		NA	ATCC 6358	Lesion

^*a*^ “NA”, not available.

^*b*^ Empty cells under Source column are either ATCC or NCTC numbers.

The potential impact of temperature on aBL effectiveness could be a factor to be considered for possible applications. Based on the results obtained in the current study, with some exceptions, the temperature did not seem to significantly impact the results of aBL on tested bacteria. The viability reductions of *Salmonella* and *Cronobacter* on PBS suspension, as dried cells on stainless steel, and on fruit surfaces were approximately 1–1.5 Log CFU with 1,512–1,944 J/cm^2^ at both temperatures tested. A couple of previous studies also observed no temperature difference in the reduction of *E. coli* O157:H7, *Salmonella* Typhimurium, *L. monocytogenes*, and *S. aureus* in orange juice (20), as well as *L. monocytogenes* on enoki mushroom (11). Furthermore, the inactivation of *L. monocytogenes* on cantaloupe rinds was not influenced by the temperature difference between 4°C and room temperature (32). However, another report observed that *Salmonella* Typhimurium in TSB was more susceptible at 10°C and 15°C than at 20°C when exposed to 461 nm LED (20). This difference in the inactivation of food-related bacteria between the current study and those reports suggests that, in addition to temperature, other parameters such as species, food matrices, and experimental conditions are quite relevant.

To ensure uniform exposure of bacterial cells to aBL within the liquid suspension, it was crucial to distribute the light evenly for accurate and reliable experimental results. Evenly distributed exposure was attained by incorporating a small magnetic stirrer into the well and placing the stir plate on the bottom. This configuration provided continuous agitation of the sample during light exposure, preventing it from settling at the bottom of the well and ensuring consistent light exposure for all cells. The results by Chen and coworkers supported the effectiveness of this experimental design in achieving uniform light distribution by comparing it with previous studies (24). They speculated that the small reductions of *E. coli*, *L. monocytogenes*, *Salmonella* Typhimurium, *Pseudomonas aeruginosa*, and *S. aureus* in their study were due to the absence of constant agitation of the liquid suspensions, which enabled uniform exposure to aBL treatment when compared with other similar studies which used magnetic stirrers (23, 33). This continuous agitation can help maintain homogeneity in the sample, which is crucial for accurate and reliable experimental results.

Previous studies evaluating the bactericidal effects of blue light on foodborne pathogens have often been limited by their focus on individual strains or species, hindering the comprehensive assessment of blue light’s effectiveness across a broader range of food-relevant bacteria. Our research aimed to address this limitation by systematically comparing the susceptibility of various foodborne pathogens and spoilage bacteria to blue light treatment. Our findings, in accordance with those of Murdoch et al. (23) and Dos Anjos et al. (34), indicated that *Salmonella* strains are generally more resistant to 405 nm blue light than other tested bacteria, such as *Staphylococcus*, *Escherichia*, *Pseudomonas*, and *Mycobacterium*. Individual strains of six foodborne pathogens, *Bacillus cereus*, *L. monocytogenes*, *S. aureus*, *E. coli* O157:H7, *P. aeruginosa*, and *Salmonella* Typhimurium, were tested under 460 nm blue light (35). More recently, Chen et al. (24) evaluated individual strains of five foodborne pathogens*—E. coli* O157:H7, *L. monocytogenes*, *P. aeruginosa*, *Salmonella* Typhimurium, and *S. aureus*—in thin liquid suspension under 405 nm blue light. They also reported a significant reduction in viable cells of *E. coli* O157:H7 and *L. monocytogenes* on stainless steel coupons using 405 nm blue light, indicating a relatively higher reduction with less dosage. Our study is one of the first studies to systematically compare the bactericidal effects of blue light against multiple food-relevant bacteria species with different strains using diverse matrices.

Unlike UV, the antimicrobial mechanism of action of aBL is thought to be similar to the general response of bacterial cells to visible light. It is hypothesized that high doses of aBL primarily cause bactericidal effects by exciting endogenous photosensitizing molecules, such as porphyrins and flavins, which subsequently generate ROS like singlet oxygen, hydroxyl radicals, and hydrogen peroxide. These ROS can induce oxidative stress by interacting with intracellular components, including nucleic acids and proteins, ultimately leading to bacterial cell death (15, 36). An interesting finding from Wang’s group was the rapid degradation of flavins into lumichrome after exposure to 405 nm blue light, suggesting a potential role in the photoinactivation process in bacteria that do not contain porphyrins (37). A recent study highlighted the pivotal role of ROS in the enhanced cytotoxic effects of pulsed aBL against *E. coli* (38). While these observations may provide insights regarding the potential antimicrobial efficacy of 405 nm light, further research is needed to fully elucidate the underlying mechanisms of inactivation.

The role of the texture of food surfaces on the effectiveness of aBLs treatment has not been investigated. Several studies have used different wavelengths of aBL to evaluate diverse food matrices (11, 28, 31, 33, 39, 40). This study used avocados and cherry tomatoes to study the impact of aBL on rough and smooth fruit surfaces. While bacterial species played a more significant role in the results, the type of food surface did not appear to be a major factor. For instance, 648 J/cm^2^ (405 nm) on fruit samples caused approximately 2.5 and 4 Log CFU population decrease on *L. monocytogenes* and *Pseudomonas* at 4°C, respectively. Similarly, the viability of *L. monocytogenes* and *P. fluorescens* on packed sliced cheese under 460–470 nm blue light (604.8 J/cm^2^) was reduced by more than 5 and 3 Log CFU at 4°C, respectively (41). The effectiveness of blue light on mango surfaces was observed with different bacterial species (40). Thus, these findings suggest that bacterial characteristics exert a greater influence on the antibacterial effect than food surface topography.

It should be noted that relatively prolonged exposure times (up to 18 h) for fruit matrices were used in our study to deliver and evaluate high doses (1,944 J/cm^2^). We intentionally used low-intensity lamps (33 mW/cm^2^) instead of high-intensity aBL lamps because the latter caused the darkening of avocado skins (data not shown). This longer exposure time with low-intensity conditions would be more suitable for fresh fruits or produce packing facilities to maintain food quality as well as effectively decontaminate fresh produce and ready-to-eat food (11, 42). Overall, exposure to 405 nm resulted in various reductions of seven bacterial genera on different testing conditions, with the susceptibility decreasing in the following order: *Pseudomonas* > Shiga toxin-producing *E. coli* > *Staphylococcus* > *Listeria* > *Lactobacillus* > *Salmonella* > *Cronobacter*.

## MATERIALS AND METHODS

### Bacterial strains and growth conditions

Four to five strains of each seven selected bacteria that were used in the study are presented in Table 1. Strains were obtained from the Center for Food Safety at the University of Georgia culture collection. Each strain was retrieved from frozen stock cultures from −80°C in 15% glycerol by transferring to tryptic soy agar (TSA; Neogen, Inc., Baltimore, MD) or to de Man, Rogosa, and Sharpe agar (MRS; BD Difco, Laboratories, Sparks, MD; for *Lactobacillus*) agar plates, followed by incubation at 37°C for 24 h for twice consecutively. Final plates were then prepared for working cultures by transferring a single colony to a sterile test tube containing TSB (Neogen, Inc., Baltimore, MD) or MRS broth (for *Lactobacillus*), followed by incubation at 37°C for 24 h and kept at 4°C. Each working culture was used for inoculation for each experiment. One milliliter of each of four to five strains was spread onto TSA or MRS agar (for *Lactobacillus*) and incubated at 37°C for 24 h. Bacterial lawns from each plate culture were scrapped with a sterile cell lifter after adding 5 mL of 0.1% buffered peptone water (BPW). Scraped cell suspensions from plates were combined by mixing 3 mL of each suspension and diluted with 0.1% BPW to obtain an average of 8 Log CFU/mL. Cocktails for each pathogen or spoilage bacteria were used to inoculate liquid, stainless steel, and food matrices, respectively.

### Blue light source

Two types of commercially available 405 nm LED lamps were used: 405 nm low-intensity blue light (405 nm, 20 W, Fasttobuy) and 405 nm high-intensity blue light (LED line 500, Honle UV technology, Gilching, Germany). Each blue light was directly placed on top of the surfaces of samples at an appropriate distance to deliver the target irradiance that was measured by LED-UV Meter (UV Meter/LED-UV Meter, Honle UV technology, Gilching, Germany). The emission dosage applied to samples was presented as Joules per square centimeter by multiplying irradiance (W/cm^2^) at the surface of samples and time in seconds (28).


E=Pt,

*E* = dose of energy density (J/cm^2^)

*P* = irradiance of power density (W/cm^2^)

*t* = time (s).

### Blue light treatment of liquid bacterial suspensions

Strain cocktail suspensions (0.1 mL) were mixed with 1.9 mL of either PBS or TSB in sterile 24-well polystyrene microplates. All independent bacterial suspensions in microplates without a lid were placed directly under 405 nm low-intensity blue light lamps at appropriate distances to receive an average irradiance of 70 mW/cm^2^. Magnetic stirrer bars (5 mm) were used to maintain homogeneous bacterial suspensions at 20 rpm within the wells and ensure consistent light exposure throughout the experiments, and a stir plate was placed under the well plates. Cell suspensions were sampled right before aBL treatment time 0 (control) and after 2 h and 6 h aBL treatment. Two temperatures were tested, 4°C and 20°C (±1.0°C, room temperature). To determine viability counts after 2 h and 6 h of aBL treatment, bacterial suspensions were serially diluted in 0.1% BPW and plated onto TSA or MRS agar (for *Lactobacilli*), followed by incubation at 37°C for up to 48 h.

### Blue light treatment of dried cells

The final inoculation concentrations were prepared as mentioned in the section “aBL treatment on stainless steel as dried cells,” targeting 7.5 Log CFU/coupon. SS coupons were washed with deionized water and sterilized by autoclaving them for 15 min before each use. For dry cells, sterilized SS coupons were spot inoculated with 100 µL and allowed to dry overnight in a biosafety cabinet. SS coupons with dry cells were placed directly under the high-intensity blue light lamp at appropriate distances to deliver an average of 161 mW/cm^2^. SS coupons were sampled right before aBL treatment time 0 (control) and after 1.5 h and 3 h aBL treatment. Because of the generation of the high-intensity lamp, the temperatures of SS coupons were 17°C ± 3°C and 41.5°C ± 1.5°C, as measured by temperature probe. At each time point, dried cell coupons were transferred to sterile sampling bags, containing 9 mL of BPW, and ultrasonicated for 10 min. Released cells from SS coupons were spread onto TSA or MRS agar (for *Lactobacilli*) and enumerated at 37°C for up to 48 h.

### Blue light treatment of surfaces of fruits

Avocados and cherry tomatoes were purchased from local supermarkets (Griffin, GA) and kept in a refrigerator (4°C). For sterilization, fruits were wiped with 70% ethanol and submerged into 2% sodium hypochlorite for 30 min, followed by rinsing with sterile deionized water twice. The surfaces of fruits were inoculated with 20 µL strain cocktails targeting 7–8 Log CFU/sample and dried overnight. Inoculated fruits were placed directly under the low-intensity blue light lamp at appropriate distances to deliver an average irradiation of 30 mW/cm^2^ for up to 18 h. Fruits were sampled right before aBL treatment time 0 (control) and after 6 h and 18 h aBL treatment. Two temperatures were tested, 4°C and 20°C (±1°C). Individual cherry tomatoes and aseptically sliced avocado surfaces were transferred to sterile sampling bags, containing 9 mL of 0.1% BPW, and stomached for 1 min. The resulting contents were serially diluted and spread onto TSA or MRS agar (for *Lactobacillus*) plates, followed by incubation at 37°C for up to 48 h.

### Statistical analysis

After each enumeration, viable colonies from TSA or MRS agar were counted with an automatic colony counter and calculated as Log CFU from three biological replications and three independent experiments on different days. Viability reductions were calculated based on pre-exposure initial counts as controls (time 0). ANOVA, along with post hoc tests (Tukey’s test) and 95% confidence intervals, was conducted to determine significant differences at a level of *P* < 0.05 probability.
